# Stroud General Hospital

**Published:** 1890-05-10

**Authors:** 


### The Hospital Workshop.

(BY OUR SPECIAL COMMISSIONER.)

### XXVI.—STROUD GENERAL HOSPITAL.

This institution, rebuilt in 1875, was originally founded in 1790, so that the present year constitutes its centenary. In order to celebrate that event the committee is making a special effort to raise £1,000 towards enlarging the building, and a further sum of £350 to bring the endowment fund up to £10,000. As pointed out in the report, additional accommodation is certainly needed for the nurses, and more room wanted "for stores and other indispensable offices." As to site, the hospital is in the highest part of the town, overlooking a valley famous for its beauty. The Stroud district is the centre of the West of England cloth trade, and contains a number of scattered villages and boroughs, the population of which amounts to some fifty or sixty thousand. The cases treated are chiefly surgical, a hundred and ninety-eight having been admitted during the year 1889, as against seventy-one medical. The proportion of accidents is comparatively small, for the machinery used in the cloth mills rarely inflicts anything beyond trifling injuries. There do not seem to be any particular complaints associated with the prevailing industry; no woolsorters' disease, for instance; but on the other hand a large amount of respiratory mischief is recorded, and this may be to some extent caused by the irritant dust of the factories. Rheumatism is extremely common, and also anæmia, especially amongst the women, both maladies having a probable connection with the somewhat foggy and malarious nature of the valley. Patients are admitted by subscribers' tickets, subject to certain conditions, such as fitness of case and the existence of bed space. There is a staff of four nurses, three for day and one for night duty, under charge of a matron, and also a resident house surgeon, Mr. R. H. Mills-Roberts, through whose courtesy the writer has been enabled to furnish the present account.

The hospital is a handsome modern building of stone. Right and left of the entrance-hall lie the house-surgeon's rooms. On the ground floor are placed the surgery, dispensary, out-patient and residential rooms, with kitchen and other domestic offices flanking one of the wings. Opposite the main door is a lift for taking helpless patients to the upper floor, where the operating-room and the wards are situated. In the corridor upstairs stands a bookcase containing the hospital library. Further along is a row of fire-buckets, filled with water in case of emergency; and near by is a shoot for dirty linen, an arrangement which is at the same time a convenience and a safeguard. There are thirty beds in all, distributed between two large and two small wards. The latter are cheerful and well lighted, with deal floors, and walls distempered in soft grey. The bedsteads are of iron, and have a curved iron rod with lifting chain at the head. They are fitted with patent canvas sacking, and hair mattrasses; while the red counterpanes throw an air of comfortable warmth over the place.

On the male side was a lad of nineteen, a printer by trade, whose case presents some features of considerable interest. He was admitted as an in-patient last October (nearly five months since), complaining of pain at the pit of the stomach, and showing a temperature normal in the mornings, but rising at night to 101° F. and sometimes to 102° F. For this increase there was no discoverable cause; no tumour, no tenderness, no phthisis, no inflammatory disorder, or other disturbance of internal organs. The patient has been kept under observation, and the history shows that during November the temperature was for the most part normal, though it rose now and then to 100° F. During December, the nightly average was about 99° F., occasionally 100° F., and on the 30th the thermometer showed 101° F. Temperature remained normal through January, with the exception of the 25th, when it reached 99·6° F., and 101° F. on the 31st. It then sank to about normal until February 13th, when it suddenly ran up to 100° F., and next day to 101° F. Since that time it has been fluctuating between 99° F. and 101° F., having mounted on one occasion to 102° F. All this while nothing has transpired to throw any fresh light on the case. It is curious to note that even when the fever is at its highest the boy feels no inconvenience or discomfort—a history, however, which tallies with the ordinary experience of these erratic temperatures. It is possible that in such cases there may be a peculiarly sensitive state of the centre in the brain that regulates the body-heat: but as yet no scientific explanation has been given of this strange condition. Its occurrence is well established, but at the same time it is practically useful to remember that some cases of so called hyperpyrexia have been shown to depend on faulty thermometers, and others to patients who have found a way of shaking up the index. Of course, a careful observer guards himself against fallacies of this kind.

A boy of sixteen, who works on the railway, has been here for some time, suffering from a series of severe complications following the kick of a horse upon his shin. The first bad symptom was *lymphangitis*, or inflammation of the lymphatics, which spread up the leg and involved the glands in the popliteal space at the back of the knee. Abscess followed, and although the important structures in the anatomical space mentioned, such as main artery, nerves, and veins, seem to have escaped, as well as the joint itself, yet the inflammation has caused necrosis, or death of a portion of the thigh bone. A boy of ten in the same ward has been in for two months with glandular abscess. He has a family history of consumption, both father and mother having died of that disease. His father, so he says, was a bricklayer, and lived in Stroud. He himself stays now with his grandmother and a sister. A large pile of boys' journals lies by the bedside, kept there for the sake of the pictures. "Yes, sir; I went to school once, but I didn't bide long enough to learn the way to read." To-day the sun has been bright and warm, so he has been allowed out for a couple of hours. He is now sitting up contentedly in bed, busy at work rolling bandages. Another curious case is what is called "late rickets." A lad of sixteen, hitherto strong and healthy, has had a rapid curving of the left leg. It was first noticed by his father some two months ago, and the deformity has been increasing further since admission, although the limb has been kept fixed in a splint.

The Report for 1889 shows a falling off of £20 in annual subscriptions. At the same time, it is pleasing to note that there has been an increase of £127 4s. 8d. in the contributions from the various mills and workshops of the neighbourhood. Out of a total of £400 19s. 2d. subscribed in this way by workpeople, £113 7s. 3d. came from one firm and £57 14s. 6d. from another. Amongst the donations is mentioned that of a poor woman, who would not give her name, but who left with the matron a sum of two pounds, made up chiefly in copper. Gifts in kind, of old linen, periodicals, fruit, vegetables, flowers, and other articles seem to have been abundant. There does not appear to be any systematic inquiry into the means of applicants, but there is not the same likelihood of abuse of charity, so much complained of in the large towns. A notice is appended to the recommendation paper: "It having been ascertained that tickets have been used by persons not entitled, by poverty, to the benefits of this Institution, the Committee will feel obliged to subscribers if they will investigate the circumstances of all applicants, so that their charity may not be abused." Some such request is made by most committees, but past experience seems to show that inquiry is a task which cannot be safely left to outside energies. One good rule requires that governors recommending as an in-patient any resident domestic servant will be required to pay one shilling and sixpence a day to defray the cost of the Board.

